# 3-(4-Acetoxy­phen­yl)-4-oxo-4*H*-1-benzopyran-5,7-diyl diacetate

**DOI:** 10.1107/S1600536809043670

**Published:** 2009-10-28

**Authors:** Huan-Qiu Li, Yin Luo, Dong-Dong Li, Hai-Liang Zhu

**Affiliations:** a School of Life Science, Nanjing University, Nanjing 210093, People’s Republic of China

## Abstract

In the title mol­ecule, C_21_H_16_O_8_, the dihedral angle between the ring systems is 58.5 (1)°. In the crystal, C—H⋯O inter­actions help to establish the packing.

## Related literature

For background to genistein derivatives, see: Li *et al.* (2006 ▶). For reference structural data, see: Allen *et al.* (1987 ▶). For related literature, see: Liu & Zhu (2005 ▶).
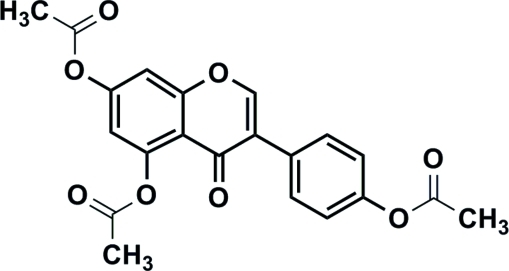


## Experimental

### 

#### Crystal data

C_21_H_16_O_8_
*M* *_r_* = 396.34
Triclinic, 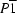

*a* = 7.6144 (14) Å
*b* = 10.6755 (19) Å
*c* = 12.533 (2) Å
α = 72.489 (3)°
β = 73.848 (3)°
γ = 74.762 (3)°
*V* = 915.2 (3) Å^3^
*Z* = 2
Mo *K*α radiation
μ = 0.11 mm^−1^
*T* = 298 K
0.30 × 0.20 × 0.10 mm

#### Data collection

Enraf–Nonius CAD-4 diffractometer
Absorption correction: ψ scan (North *et al.*, 1968 ▶) *T*_min_ = 0.967, *T*_max_ = 0.989
4847 measured reflections
3043 independent reflections
1968 reflections with *I* > 2σ(*I*)
*R*_int_ = 0.025
200 standard reflections every 3 reflections intensity decay: 1%

#### Refinement

*R*[*F* ^2^ > 2σ(*F* ^2^)] = 0.046
*wR*(*F* ^2^) = 0.113
*S* = 0.96
3043 reflections
265 parameters
H-atom parameters constrained
Δρ_max_ = 0.17 e Å^−3^
Δρ_min_ = −0.17 e Å^−3^

### 

Data collection: *CAD-4 Software* (Enraf–Nonius, 1989 ▶); cell refinement: *CAD-4 Software*; data reduction: *XCAD4* (Harms & Wocadlo, 1995 ▶); program(s) used to solve structure: *SHELXS97* (Sheldrick, 2008 ▶); program(s) used to refine structure: *SHELXL97* (Sheldrick, 2008 ▶); molecular graphics: *SHELXTL* (Sheldrick, 2008 ▶); software used to prepare material for publication: *SHELXTL*.

## Supplementary Material

Crystal structure: contains datablocks global, I. DOI: 10.1107/S1600536809043670/hb5164sup1.cif

Structure factors: contains datablocks I. DOI: 10.1107/S1600536809043670/hb5164Isup2.hkl

Additional supplementary materials:  crystallographic information; 3D view; checkCIF report

## Figures and Tables

**Table 1:** Hydrogen-bond geometry (Å, °)

*D*—H⋯*A*	*D*—H	H⋯*A*	*D*⋯*A*	*D*—H⋯*A*
C7—H7⋯O8^i^	0.93	2.47	3.396 (3)	175
C19—H19*A*⋯O7^ii^	0.96	2.57	3.523 (3)	173
C21—H21*B*⋯O3^iii^	0.96	2.44	3.328 (3)	154

Symmetry codes: (i) 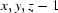; (ii) 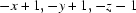; (iii) 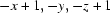.

**Fig. 1.**
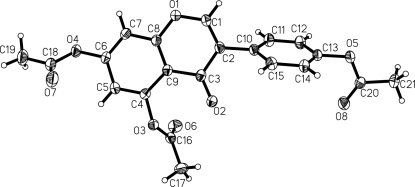
The structure of (I) showing 30% probability displacement ellipsoids.

## supplementary crystallographic information

### Comment

Genistein derivatives have been pharmacologically shown some biological activitites (Li *et al.*, 2006). In the title compound, (I) (Fig. 1), the bond lengths and angles (Table 1) are within normal ranges (Allen *et al.*, 1987). The dihedral angle between the least-squares planes of the two benzene rings is 123.8 °. The crystal packing is stabilized by van der Waals forces.

### Experimental

Genistein (0.41 g, 1.5 mmol), iodomethane (0.62 ml, 6 mmol) and potassium carbonate (0.42 g, 3 mmol) in 50 ml of dry acetone were sonicated. After the completion of reaction, the given mixture was cooled to room temperature followed by filtration. The filtrate was distilled to give a yellow solid. They were washed with aqueous saturated sodium bicarbonate twice. The solid was dissolved in acetone (15 ml) and stirred for about 10 min to give a clear solution. After keeping the solution in air for 10 d, colorless block-shaped crystals of (I) were formed at the bottom of the vesssl on slow evaporation of the solvent.They were collected, washed three times with acetone and dried in a vacuum desiccator using CaCl_2_. The compound was isolated in 98% yield.

### Refinement

All H atoms were positioned geometrically (C—H = 0.93 Å for the aromatic H atoms and C—H = 0.96 Å for the aliphatic H atoms) and were refined as riding, with *U*_iso_(H) = 1.2*U*_eq_(C) and *U*_iso_(H) = 1.2*U*_eq_(N).

### Crystal data

**Table:**

C_21_H_16_O_8_	*Z* = 2
*M**_r_* = 396.34	*F*(000) = 412
Triclinic, *P*1	*D*_x_ = 1.438 Mg m^−^^3^
Hall symbol: -P 1	Mo *K*α radiation, λ = 0.71073 Å
*a* = 7.6144 (14) Å	Cell parameters from 25 reflections
*b* = 10.6755 (19) Å	θ = 9–12°
*c* = 12.533 (2) Å	µ = 0.11 mm^−^^1^
α = 72.489 (3)°	*T* = 298 K
β = 73.848 (3)°	Block, colourless
γ = 74.762 (3)°	0.30 × 0.20 × 0.10 mm
*V* = 915.2 (3) Å^3^	

### Data collection

**Table:**

Enraf–Nonius CAD-4 diffractometer	1968 reflections with *I* > 2σ(*I*)
Radiation source: fine-focus sealed tube	*R*_int_ = 0.025
graphite	θ_max_ = 25.0°, θ_min_ = 1.8°
ω/2θ scans	*h* = −8→9
Absorption correction: ψ scan (North *et al.*, 1968)	*k* = −12→11
*T*_min_ = 0.967, *T*_max_ = 0.989	*l* = −14→14
4847 measured reflections	200 standard reflections every 3 reflections
3043 independent reflections	intensity decay: 1%

### Refinement

**Table:**

Refinement on *F*^2^	Primary atom site location: structure-invariant direct methods
Least-squares matrix: full	Secondary atom site location: difference Fourier map
*R*[*F*^2^ > 2σ(*F*^2^)] = 0.046	Hydrogen site location: inferred from neighbouring sites
*wR*(*F*^2^) = 0.113	H-atom parameters constrained
*S* = 0.96	*w* = 1/[σ^2^(*F*_o_^2^) + (0.0486*P*)^2^] where *P* = (*F*_o_^2^ + 2*F*_c_^2^)/3
3043 reflections	(Δ/σ)_max_ = 0.008
265 parameters	Δρ_max_ = 0.17 e Å^−^^3^
0 restraints	Δρ_min_ = −0.17 e Å^−^^3^

### Special details

**Table:**

Geometry. All e.s.d.'s (except the e.s.d. in the dihedral angle between two l.s. planes) are estimated using the full covariance matrix. The cell e.s.d.'s are taken into account individually in the estimation of e.s.d.'s in distances, angles and torsion angles; correlations between e.s.d.'s in cell parameters are only used when they are defined by crystal symmetry. An approximate (isotropic) treatment of cell e.s.d.'s is used for estimating e.s.d.'s involving l.s. planes.
Refinement. Refinement of *F*^2^ against ALL reflections. The weighted *R*-factor *wR* and goodness of fit *S* are based on *F*^2^, conventional *R*-factors *R* are based on *F*, with *F* set to zero for negative *F*^2^. The threshold expression of *F*^2^ > σ(*F*^2^) is used only for calculating *R*-factors(gt) *etc*. and is not relevant to the choice of reflections for refinement. *R*-factors based on *F*^2^ are statistically about twice as large as those based on *F*, and *R*- factors based on ALL data will be even larger.

### Fractional atomic coordinates and isotropic or equivalent isotropic displacement parameters (Å^2^)

**Table:**

	*x*	*y*	*z*	*U*_iso_*/*U*_eq_	
C1	0.2549 (3)	−0.0952 (3)	0.18430 (19)	0.0501 (6)	
H1	0.2405	−0.1833	0.2186	0.060*	
C2	0.2866 (3)	−0.0287 (2)	0.24896 (17)	0.0396 (6)	
C3	0.3137 (3)	0.1090 (2)	0.19959 (17)	0.0384 (6)	
C4	0.2951 (3)	0.2985 (2)	0.01826 (17)	0.0359 (5)	
C5	0.2684 (3)	0.3459 (2)	−0.09068 (17)	0.0400 (6)	
H5	0.2705	0.4350	−0.1289	0.048*	
C6	0.2379 (3)	0.2591 (2)	−0.14434 (17)	0.0396 (6)	
C7	0.2302 (3)	0.1288 (2)	−0.08973 (17)	0.0419 (6)	
H7	0.2095	0.0718	−0.1263	0.050*	
C8	0.2545 (3)	0.0838 (2)	0.02250 (17)	0.0397 (6)	
C9	0.2890 (3)	0.1646 (2)	0.08047 (17)	0.0353 (5)	
C10	0.2867 (3)	−0.0925 (2)	0.37225 (18)	0.0401 (6)	
C11	0.1331 (3)	−0.1409 (3)	0.44474 (19)	0.0489 (6)	
H11	0.0303	−0.1346	0.4158	0.059*	
C12	0.1296 (3)	−0.1986 (3)	0.55972 (19)	0.0494 (6)	
H12	0.0259	−0.2315	0.6081	0.059*	
C13	0.2823 (3)	−0.2065 (2)	0.60117 (18)	0.0433 (6)	
C14	0.4364 (3)	−0.1589 (2)	0.53217 (18)	0.0463 (6)	
H14	0.5384	−0.1655	0.5620	0.056*	
C15	0.4382 (3)	−0.1008 (2)	0.41701 (19)	0.0468 (6)	
H15	0.5417	−0.0671	0.3694	0.056*	
C16	0.2072 (3)	0.4303 (2)	0.15330 (19)	0.0408 (6)	
C17	0.2872 (4)	0.5041 (3)	0.2056 (2)	0.0600 (7)	
H17A	0.1881	0.5542	0.2518	0.090*	
H17B	0.3570	0.5643	0.1462	0.090*	
H17C	0.3680	0.4415	0.2527	0.090*	
C18	0.3122 (4)	0.3839 (3)	−0.33891 (19)	0.0513 (7)	
C19	0.2309 (4)	0.4375 (3)	−0.4425 (2)	0.0703 (8)	
H19A	0.3218	0.4743	−0.5066	0.105*	
H19B	0.1232	0.5063	−0.4291	0.105*	
H19C	0.1953	0.3665	−0.4588	0.105*	
C20	0.2196 (3)	−0.1878 (3)	0.79073 (19)	0.0423 (6)	
C21	0.2206 (3)	−0.2675 (3)	0.91124 (17)	0.0501 (7)	
H21A	0.1991	−0.2079	0.9597	0.075*	
H21B	0.3393	−0.3267	0.9141	0.075*	
H21C	0.1240	−0.3191	0.9373	0.075*	
O1	0.2418 (2)	−0.04736 (16)	0.07344 (12)	0.0517 (5)	
O2	0.3515 (2)	0.17371 (16)	0.25336 (13)	0.0543 (5)	
O3	0.3377 (2)	0.38639 (15)	0.06600 (12)	0.0400 (4)	
O4	0.1996 (2)	0.30585 (17)	−0.25361 (12)	0.0496 (4)	
O5	0.2817 (2)	−0.26831 (16)	0.71821 (12)	0.0491 (4)	
O6	0.0523 (2)	0.41095 (18)	0.17983 (14)	0.0587 (5)	
O7	0.4524 (3)	0.4012 (2)	−0.32773 (14)	0.0704 (6)	
O8	0.1702 (2)	−0.06907 (19)	0.76094 (14)	0.0620 (5)	

### Atomic displacement parameters (Å^2^)

**Table:**

	*U*^11^	*U*^22^	*U*^33^	*U*^12^	*U*^13^	*U*^23^
C1	0.0721 (17)	0.0365 (15)	0.0389 (14)	−0.0123 (13)	−0.0165 (12)	0.0003 (11)
C2	0.0428 (13)	0.0343 (14)	0.0396 (13)	−0.0042 (11)	−0.0109 (11)	−0.0070 (11)
C3	0.0342 (12)	0.0426 (15)	0.0383 (12)	−0.0040 (11)	−0.0100 (10)	−0.0108 (11)
C4	0.0325 (12)	0.0384 (14)	0.0384 (13)	−0.0076 (10)	−0.0090 (10)	−0.0100 (10)
C5	0.0410 (13)	0.0381 (14)	0.0386 (13)	−0.0093 (11)	−0.0085 (10)	−0.0049 (11)
C6	0.0407 (13)	0.0469 (16)	0.0314 (12)	−0.0075 (11)	−0.0085 (10)	−0.0101 (11)
C7	0.0486 (14)	0.0438 (16)	0.0386 (13)	−0.0120 (12)	−0.0096 (11)	−0.0152 (11)
C8	0.0431 (14)	0.0363 (15)	0.0372 (13)	−0.0069 (11)	−0.0075 (10)	−0.0073 (10)
C9	0.0329 (12)	0.0344 (14)	0.0372 (12)	−0.0050 (10)	−0.0075 (10)	−0.0082 (10)
C10	0.0490 (14)	0.0318 (14)	0.0376 (13)	−0.0028 (11)	−0.0114 (11)	−0.0083 (10)
C11	0.0512 (15)	0.0510 (17)	0.0447 (14)	−0.0123 (13)	−0.0168 (12)	−0.0041 (12)
C12	0.0512 (15)	0.0505 (17)	0.0421 (14)	−0.0154 (12)	−0.0050 (12)	−0.0049 (12)
C13	0.0594 (16)	0.0364 (15)	0.0332 (12)	−0.0075 (12)	−0.0129 (11)	−0.0064 (10)
C14	0.0513 (15)	0.0454 (16)	0.0429 (14)	−0.0091 (12)	−0.0181 (12)	−0.0049 (12)
C15	0.0486 (15)	0.0447 (16)	0.0452 (14)	−0.0128 (12)	−0.0095 (12)	−0.0058 (12)
C16	0.0457 (15)	0.0366 (15)	0.0408 (13)	−0.0067 (11)	−0.0142 (11)	−0.0071 (11)
C17	0.0672 (18)	0.0626 (19)	0.0603 (16)	−0.0176 (15)	−0.0137 (14)	−0.0256 (14)
C18	0.0628 (18)	0.0495 (17)	0.0384 (14)	−0.0102 (14)	−0.0084 (13)	−0.0095 (12)
C19	0.088 (2)	0.071 (2)	0.0444 (15)	−0.0077 (16)	−0.0230 (15)	−0.0021 (14)
C20	0.0391 (13)	0.0459 (17)	0.0446 (14)	−0.0077 (12)	−0.0108 (11)	−0.0139 (12)
C21	0.0490 (14)	0.0631 (18)	0.0385 (13)	−0.0107 (13)	−0.0128 (11)	−0.0100 (12)
O1	0.0822 (12)	0.0373 (10)	0.0410 (9)	−0.0177 (9)	−0.0192 (8)	−0.0071 (7)
O2	0.0783 (12)	0.0447 (11)	0.0495 (10)	−0.0160 (9)	−0.0333 (9)	−0.0042 (8)
O3	0.0431 (9)	0.0406 (10)	0.0403 (8)	−0.0140 (7)	−0.0096 (7)	−0.0102 (7)
O4	0.0595 (10)	0.0558 (11)	0.0355 (9)	−0.0138 (9)	−0.0180 (8)	−0.0050 (8)
O5	0.0678 (11)	0.0410 (10)	0.0363 (9)	−0.0091 (8)	−0.0147 (8)	−0.0043 (8)
O6	0.0420 (10)	0.0676 (13)	0.0711 (12)	−0.0153 (9)	−0.0061 (9)	−0.0254 (10)
O7	0.0757 (13)	0.0925 (16)	0.0483 (11)	−0.0369 (12)	−0.0087 (10)	−0.0113 (10)
O8	0.0805 (13)	0.0464 (12)	0.0555 (11)	0.0031 (10)	−0.0225 (10)	−0.0136 (9)

### Geometric parameters (Å, °)

**Table:**

C1—C2	1.328 (3)	C13—C14	1.366 (3)
C1—O1	1.351 (2)	C13—O5	1.415 (2)
C1—H1	0.9300	C14—C15	1.387 (3)
C2—C3	1.459 (3)	C14—H14	0.9300
C2—C10	1.490 (3)	C15—H15	0.9300
C3—O2	1.227 (2)	C16—O6	1.189 (2)
C3—C9	1.477 (3)	C16—O3	1.365 (3)
C4—C5	1.359 (3)	C16—C17	1.485 (3)
C4—O3	1.396 (2)	C17—H17A	0.9600
C4—C9	1.414 (3)	C17—H17B	0.9600
C5—C6	1.392 (3)	C17—H17C	0.9600
C5—H5	0.9300	C18—O7	1.182 (3)
C6—C7	1.362 (3)	C18—O4	1.379 (3)
C6—O4	1.394 (2)	C18—C19	1.487 (3)
C7—C8	1.391 (3)	C19—H19A	0.9600
C7—H7	0.9300	C19—H19B	0.9600
C8—O1	1.370 (3)	C19—H19C	0.9600
C8—C9	1.394 (3)	C20—O8	1.196 (3)
C10—C11	1.380 (3)	C20—O5	1.344 (3)
C10—C15	1.389 (3)	C20—C21	1.495 (3)
C11—C12	1.382 (3)	C21—H21A	0.9600
C11—H11	0.9300	C21—H21B	0.9600
C12—C13	1.374 (3)	C21—H21C	0.9600
C12—H12	0.9300		
			
C2—C1—O1	126.3 (2)	C12—C13—O5	119.0 (2)
C2—C1—H1	116.9	C13—C14—C15	118.9 (2)
O1—C1—H1	116.9	C13—C14—H14	120.5
C1—C2—C3	119.5 (2)	C15—C14—H14	120.5
C1—C2—C10	120.2 (2)	C14—C15—C10	120.5 (2)
C3—C2—C10	120.22 (19)	C14—C15—H15	119.7
O2—C3—C2	122.63 (19)	C10—C15—H15	119.7
O2—C3—C9	122.9 (2)	O6—C16—O3	122.7 (2)
C2—C3—C9	114.48 (19)	O6—C16—C17	126.9 (2)
C5—C4—O3	117.23 (19)	O3—C16—C17	110.41 (19)
C5—C4—C9	122.3 (2)	C16—C17—H17A	109.5
O3—C4—C9	120.37 (17)	C16—C17—H17B	109.5
C4—C5—C6	119.1 (2)	H17A—C17—H17B	109.5
C4—C5—H5	120.4	C16—C17—H17C	109.5
C6—C5—H5	120.4	H17A—C17—H17C	109.5
C7—C6—C5	121.76 (19)	H17B—C17—H17C	109.5
C7—C6—O4	117.3 (2)	O7—C18—O4	123.0 (2)
C5—C6—O4	120.7 (2)	O7—C18—C19	127.3 (2)
C6—C7—C8	117.9 (2)	O4—C18—C19	109.8 (2)
C6—C7—H7	121.1	C18—C19—H19A	109.5
C8—C7—H7	121.1	C18—C19—H19B	109.5
O1—C8—C7	115.1 (2)	H19A—C19—H19B	109.5
O1—C8—C9	121.63 (18)	C18—C19—H19C	109.5
C7—C8—C9	123.3 (2)	H19A—C19—H19C	109.5
C8—C9—C4	115.59 (18)	H19B—C19—H19C	109.5
C8—C9—C3	120.0 (2)	O8—C20—O5	123.6 (2)
C4—C9—C3	124.43 (19)	O8—C20—C21	125.8 (2)
C11—C10—C15	118.88 (19)	O5—C20—C21	110.6 (2)
C11—C10—C2	120.20 (19)	C20—C21—H21A	109.5
C15—C10—C2	120.9 (2)	C20—C21—H21B	109.5
C10—C11—C12	121.0 (2)	H21A—C21—H21B	109.5
C10—C11—H11	119.5	C20—C21—H21C	109.5
C12—C11—H11	119.5	H21A—C21—H21C	109.5
C13—C12—C11	118.7 (2)	H21B—C21—H21C	109.5
C13—C12—H12	120.7	C1—O1—C8	117.90 (18)
C11—C12—H12	120.7	C16—O3—C4	117.96 (15)
C14—C13—C12	121.9 (2)	C18—O4—C6	119.08 (18)
C14—C13—O5	119.02 (19)	C20—O5—C13	116.67 (18)
			
O1—C1—C2—C3	−1.0 (4)	C1—C2—C10—C15	125.7 (3)
O1—C1—C2—C10	176.5 (2)	C3—C2—C10—C15	−56.8 (3)
C1—C2—C3—O2	−176.7 (2)	C15—C10—C11—C12	−0.9 (4)
C10—C2—C3—O2	5.7 (3)	C2—C10—C11—C12	−179.1 (2)
C1—C2—C3—C9	4.2 (3)	C10—C11—C12—C13	0.3 (4)
C10—C2—C3—C9	−173.35 (19)	C11—C12—C13—C14	0.1 (4)
O3—C4—C5—C6	175.77 (18)	C11—C12—C13—O5	−178.5 (2)
C9—C4—C5—C6	−1.4 (3)	C12—C13—C14—C15	0.2 (4)
C4—C5—C6—C7	1.4 (3)	O5—C13—C14—C15	178.7 (2)
C4—C5—C6—O4	176.38 (18)	C13—C14—C15—C10	−0.8 (4)
C5—C6—C7—C8	−0.2 (3)	C11—C10—C15—C14	1.1 (4)
O4—C6—C7—C8	−175.31 (19)	C2—C10—C15—C14	179.3 (2)
C6—C7—C8—O1	178.82 (19)	C2—C1—O1—C8	−2.8 (4)
C6—C7—C8—C9	−1.1 (3)	C7—C8—O1—C1	−176.8 (2)
O1—C8—C9—C4	−178.87 (18)	C9—C8—O1—C1	3.1 (3)
C7—C8—C9—C4	1.0 (3)	O6—C16—O3—C4	−9.9 (3)
O1—C8—C9—C3	0.3 (3)	C17—C16—O3—C4	170.81 (18)
C7—C8—C9—C3	−179.8 (2)	C5—C4—O3—C16	111.1 (2)
C5—C4—C9—C8	0.3 (3)	C9—C4—O3—C16	−71.6 (2)
O3—C4—C9—C8	−176.86 (18)	O7—C18—O4—C6	8.7 (4)
C5—C4—C9—C3	−178.8 (2)	C19—C18—O4—C6	−172.2 (2)
O3—C4—C9—C3	4.0 (3)	C7—C6—O4—C18	−136.0 (2)
O2—C3—C9—C8	177.1 (2)	C5—C6—O4—C18	48.8 (3)
C2—C3—C9—C8	−3.8 (3)	O8—C20—O5—C13	−1.6 (3)
O2—C3—C9—C4	−3.8 (3)	C21—C20—O5—C13	177.94 (18)
C2—C3—C9—C4	175.25 (19)	C14—C13—O5—C20	89.1 (3)
C1—C2—C10—C11	−56.2 (3)	C12—C13—O5—C20	−92.3 (3)
C3—C2—C10—C11	121.3 (2)		

### Hydrogen-bond geometry (Å, °)

**Table:**

*D*—H···*A*	*D*—H	H···*A*	*D*···*A*	*D*—H···*A*
C7—H7···O8^i^	0.93	2.47	3.396 (3)	175
C19—H19A···O7^ii^	0.96	2.57	3.523 (3)	173
C21—H21B···O3^iii^	0.96	2.44	3.328 (3)	154

Symmetry codes: (i) *x*, *y*, *z*−1; (ii) −*x*+1, −*y*+1, −*z*−1; (iii) −*x*+1, −*y*, −*z*+1.
